# Feasibility of Using Ultra-High Field (7 T) MRI for Clinical Surgical Targeting

**DOI:** 10.1371/journal.pone.0037328

**Published:** 2012-05-17

**Authors:** Yuval Duchin, Aviva Abosch, Essa Yacoub, Guillermo Sapiro, Noam Harel

**Affiliations:** 1 Department of Radiology, Center for Magnetic Resonance Research, University of Minnesota, Minneapolis, Minnesota, United States of America; 2 Department of Neurosurgery, University of Minnesota, Minneapolis, Minnesota, United States of America; 3 Department of Electrical and Computer Engineering, University of Minnesota, Minneapolis, Minnesota, United States of America; University of California San Francisco, United States of America

## Abstract

The advantages of ultra-high magnetic field (7 Tesla) MRI for basic science research and neuroscience applications have proven invaluable. Structural and functional MR images of the human brain acquired at 7 T exhibit rich information content with potential utility for clinical applications. However, (1) substantial increases in susceptibility artifacts, and (2) geometrical distortions at 7 T would be detrimental for stereotactic surgeries such as deep brain stimulation (DBS), which typically use 1.5 T images for surgical planning. Here, we explore whether these issues can be addressed, making feasible the use of 7 T MRI to guide surgical planning. Twelve patients with Parkinson's disease, candidates for DBS, were scanned on a standard clinical 1.5 T MRI and a 7 T MRI scanner. Qualitative and quantitative assessments of global and regional distortion were evaluated based on anatomical landmarks and transformation matrix values. Our analyses show that distances between identical landmarks on 1.5 T vs. 7 T, in the mid-brain region, were less than one voxel, indicating a successful co-registration between the 1.5 T and 7 T images under these specific imaging parameter sets. On regional analysis, the central part of the brain showed minimal distortion, while inferior and frontal areas exhibited larger distortion due to proximity to air-filled cavities. We conclude that 7 T MR images of the central brain regions have comparable distortions to that observed on a 1.5 T MRI, and that clinical applications targeting structures such as the STN, are feasible with information-rich 7 T imaging.

## Introduction

The advantages of ultra-high field (7 Tesla (T) and beyond) MRI machines for basic science research and neuroscience applications, have proven to be invaluable [1], [2], [3].

Functional MRI (fMRI) studies conducted at high-fields, capitalizing on the enhanced sensitivity and specificity of the measured blood-oxygen-level-dependent (BOLD) signals, successfully demonstrated the mapping of functional organizations of neuronal architectures in cerebral cortex at unprecedented levels of details [4], [5]. Structural (i.e., anatomical) images of the human brain acquired at 7 T exhibit rich informational content with potential utility for clinical applications [6], [7], [8], [9]. Such publications have led to a continued growth of interest in high-field MRI for research and clinical applications, and continued support of 7 T product lines by the major manufacturers.

Although of obvious clinical interest, there are potential obstacles for the clinical application of 7 T MRI. In particular, those of most concern are: (1) increased power deposition, (2) the substantial increase in susceptibility artifacts, and perhaps most important for surgical applications, (3) geometrical distortion. All of these confounds increase with magnetic field strength, and can significantly compromise image acquisition, and interpretability of the images. Despite these limitations, and due to the unique contributions of 7 T MRI mentioned above, the question to be addressed is whether 7 T MRI can be of use for clinical applications.

Recently, 7 T MRI was demonstrated to improve the resolution of the internal architecture of basal ganglia and thalamic structures, including surrounding regions—a finding which is of significance for the placement of deep brain stimulating (DBS) electrodes during the treatment of certain movement and neuropsychiatric disorders [7], [8], [9]. Critical to the success of DBS surgery is the precise localization of the intended target structure for subsequent implantation of the DBS electrode. Current techniques involve stereotactic imaging combined with consensus coordinates [10] to identify a first approximation of the DBS target, which is then modified or confirmed based on intraoperative electrophysiological techniques, including microelectrode recording and macrostimulation, as well as patient behavioral feedback. However, certain DBS targets, such as the ventro-intermediate nucleus (VIM) of thalamus for the treatment of essential tremor, have not yet been visualized using standard clinical imaging. Visualization of internal thalamic nuclei is a challenging, if not impossible, task using clinically-available 1.5 or 3 T MRI systems [11], [12], [13]. The use of 7 T MRI with enhanced signal-to-noise ratio (SNR), combined with MR sequences that are more advantageous at higher magnetic field, such as susceptibility-weighted imaging (SWI) [14], [15], [16], has yielded high-resolution images with enhanced contrast, allowing visualization of internal thalamic nuclei in individual patients. However, concerns have been raised as to the validity of these images for stereotactic targeting, due to the possibility of increased distortion at high field [9].

Previous work that evaluated hardware-related geometrical distortion of 7 T and 1.5 T images, using an MRI phantom, found very low amounts of distortion in both cases [17], [18]. However, a question remaining is whether these phantom-based findings, are applicable *in vivo* with actual subject-dependent susceptibility-induced changes, and in the clinical setting where an accurate targeting of a specific brain target is required.

In this work, we sought to characterize, *in vivo*, the amount of geometrical distortion present at 7 T relative to standard clinical imaging obtained on a 1.5 T scanner, in subjects undergoing preoperative evaluations for DBS surgery. We demonstrate that the common practice for brain surgery planning, where co-registering clinical stereotactic 1.5 T MR images to CT images is done in order to correct possible geometric distortion, can be applied in a straightforward way for 7 T images. Further, we demonstrate that images acquired at 7 T exhibit minimal distortions in the midbrain region, compared to 1.5 T images, and can be corrected by piecewise (regional-based) linear registrations, thereby documenting the feasibility of using high-field MRI as a clinical tool.

## Materials and Methods

Approval for this study was obtained from the Institutional Review Board at the University of Minnesota. All subjects provided informed written consent prior to participating in the study. Subjects were derived from the population of patients undergoing preoperative evaluation for DBS surgery in the Department of Neurosurgery at the University of Minnesota Medical Center. Inclusion criteria included patients with a diagnosis of idiopathic Parkinson's disease deemed suitable candidates for DBS surgery [19]. Subjects with claustrophobia or contraindications to MR imaging were excluded from this study.

Twelve patients diagnosed with Parkinson's disease (eight men and four women) with average age 61 years and average disease duration of 10 years, were enrolled in this study. All subjects underwent scanning on both a clinical 1.5 T Philips Intera MRI system and a Siemens Somatom Sensation 16 CT scanner at the University of Minnesota Fairview Hospital, and on a 7 T MRI scanner (Magnex Scientific, UK) at the Center for Magnetic Resonance Research (CMRR) at the University of Minnesota, using T_1_-Weighted (T_1_W) and T_2_-Weighted (T_2_W) imaging protocols. Six patients were scanned with a first-generation passively shielded 7 T magnet (7T/PS), using a Siemens Avanto body gradient set capable of 40 mT/m and a maximum slew rate of 200 T/m/s, and six patients were scanned with a new actively shielded 7 T magnet (7T/AS), using SC72 gradients capable of 70 mT/m and a 200 T/m/s slew rate. Both 7 T magnets were driven by a Siemens console (Erlangen, Germany).

All 1.5 T images were acquired using a Philips Synergy Flex Interventional – Large coil. All 7 T images were acquired with a 24-element head array coil (Nova Medical, Inc, Burlington, MA) and were acquired with the MRI vendor's 3D distortion correction, which compensates for geometrical distortions originating from gradients nonlinearities.

### Acquisition parameters for 7T/PS and 7T/AS images

#### T_1_-Weighted MRI

Images with 1 mm isotropic resolution were acquired with the standard Siemens 3D MPRAGE sequence: FOV: 256×256×176 mm^3^ (256×256×176 matrix), TR = 3000 ms; TI = 1500 ms, TE = 4.03 ms (for 7T/PS), and 3.27 ms (for 7T/AS), nominal flip angle = 5°, bandwidth = 140 Hz/pxl (for 7T/PS) and 180 Hz/pxl (for 7T/AS), maximum readout gradient strength = 22 mT/m (for 7T/PS) and 40 mT/m (for 7T/AS), total acquisition time = 3.5 minutes, acceleration factor of 2 (GRAPPA) along the phase encode direction.

T_2_-Weighted MRI: An axial slab oriented parallel to the AC-PC line that runs from the top of thalamus down through the upper pons was acquired using a 2D turbo spin echo (TSE) sequence with the following image parameters: FOV: 200×200×40 mm^3^; 512×512×20 matrix (0.39×0.39×2.0 mm^3^), TR/TE 8000/58 msec, flip angle = 150°, bandwidth = 255 Hz/pxl, maximum readout gradient strength = 15.4 mT/m, acceleration factor of 3 (GRAPPA) along the phase encoding direction. The total acquisition time was 5.5 minutes for 4 averages.

### Acquisition parameters for 1.5 T images

#### T_1_-Weighted MRI

Images with 1 mm isotropic resolution were acquired using the following standard clinical protocol: FOV: 256×256×165 mm^3^; 256×256×165 matrix (1×1×1 mm), TR = 7.07 ms; TE = 3.19 ms, nominal flip angle of 8°, bandwidth of 241 Hz/pxl, and total acquisition time of ten minutes.

#### T_2_-Weighted MRI

A commercial Philips sequence *T2W TSE (Turbo Spin Echo) 5–19 CLEAR* (Multi-channel RF coil sensitivity normalization) clinical protocol was used with the following image parameters: FOV: 256×256×58 mm^2^; 512×512×29 matrix (0.5×0.5×2.0 mm^3^), TR/TE 4866/90 msec, bandwidth of 222 Hz/pxl and 10 averages, for a total acquisition time of 13 minutes.

### CT acquisition parameters

Images were acquired with 0.75 mm slice thickness, 512×512 matrix, FOV: 250×250 mm^2^ (to encompass fiducial marks), 0.0 degree gantry tilt, 120 kV, 250 mAs.

### Distortion analysis

The method used to evaluate the geometric distortion present on 7 T images relative to 1.5 T MR images was critically dependent on achieving a satisfactory co-registration between corresponding images using visual inspection, as is routinely done in clinical practice. Obtaining this adequate co-registration, together with the analysis of the transformation operator that was required for the registration, allows for both a qualitative and quantitative assessment of any deformations imposed by the different scanners be it related to magnetic field strength or scanner hardware (i.e. gradients).

**Figure 1:**
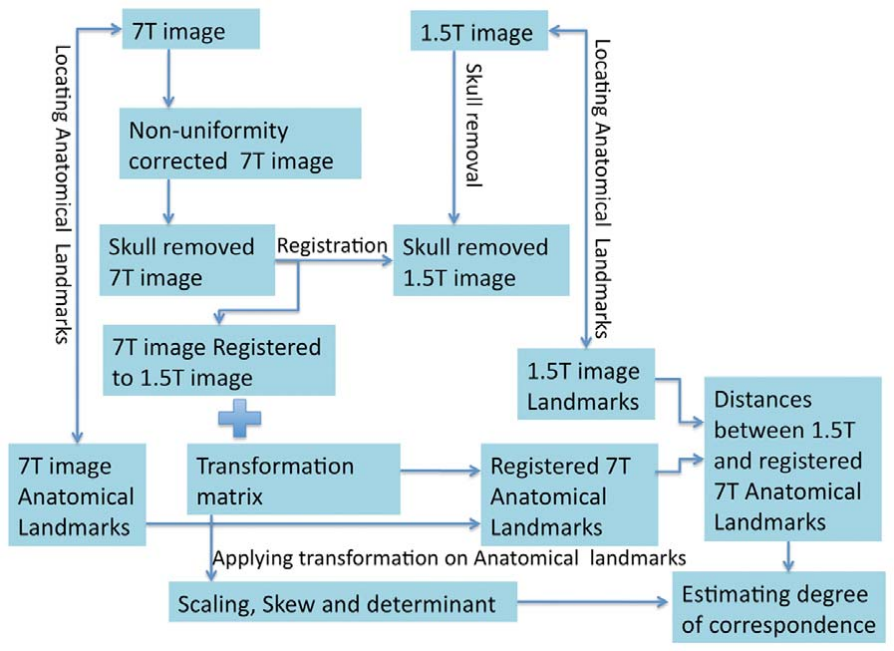
Workflow of data processing and analysis. The workflow process of image post-processing and quantitative estimation of the global degree of correspondence between co-registered 7 T and 1.5 T MR images, using anatomical landmarks method.

Figure 1 shows the workflow of the pre-processing, registration, and evaluation of the global registration quality. Initially, the T_1_W 7T MR images were corrected for non-uniformity using the FSL FAST tool [20], [21], [22]. Following the non-uniformity correction, non-brain structures (e.g., skull, neck, nose, etc.) were extracted from both 7 T and 1.5 T images using FSL's Brain Extraction Tool (BET) [23], allowing for better co-registration results. The 7 T images were registered to the 1.5 T images by a standard linear image co-registration technique, using the FSL FLIRT software [24]. The co-registrations were performed first with 6 degrees-of-freedom (DOF) and a search-angle resolution of 3 degrees for coarse search and alignment of the images (rigid rotation and translation). Following the 6 DOF co-registration, an affine 12 DOF registration was applied for fine adjustment of scaling and shearing. The cost function for the optimization process was Mutual Information (MI), a common and robust cost function that is widely used for the registration of medical images, and is routinely used for the co-registration of multiple imaging modalities [25]. This type of affine registration is fundamental, robust and is commonly used in the clinic for stereotactic surgery planning. Each registration produced an affine transformation matrix, which consists of rigid body transformations, scaling factors, and shear angles.

**Figure 2:**
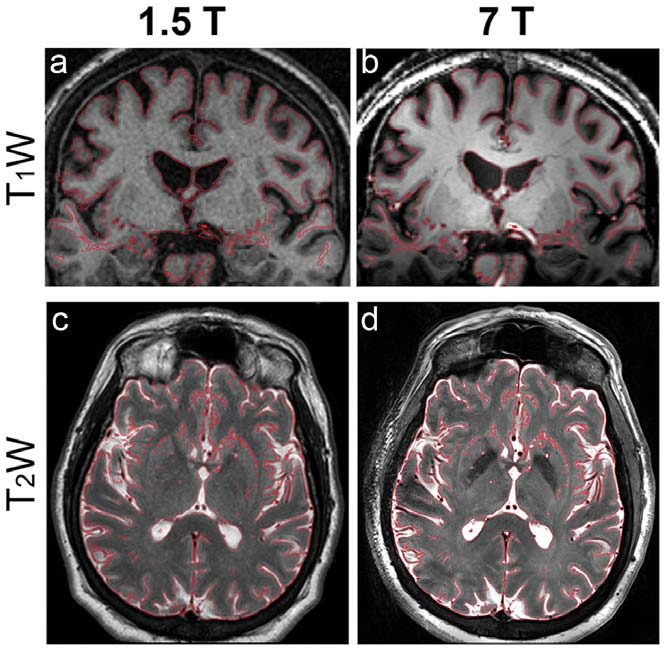
Visual example of the registration results. Edges of brain structures obtained from the registered 7 T image (right column) are superimposed on the 1.5 T image (left column). Top row: T_1_W coronal images acquired at (a) 1.5 T and (b) 7 T. Bottom row: T_2_W axial images acquired at (c) 1.5 T and (d) 7 T, respectively. Note the high degree of correspondence between the 7 T red iso-contour edge lines superimposed on the 1.5 T images, indicating minimal distortion in the 7 T images compared to the clinical 1.5 T images. This method follows the current standard practice used in the operating room, in which the surgeon overlays the registered image on top of the reference image and toggles between them in order to determine, visually, how well regional brain structures coincide in the different image sets.

We employed three methods to evaluate the degree of correspondence between the registered 7 T images and the clinical 1.5 T images. First, we used FSL's Slicer function to draw edge lines to qualitatively and visually assess the degree of image correspondence and the co-registration quality. In this method, edges obtained from the registered 7 T image were superimposed on the 1.5 T image (Figure 2). This method follows the current standard practice used in the operating room, in which the surgeon overlays the registered image(s) on top of the reference image (e.g., Medtronic Stealth Station, Framelink software, Minneapolis, MN), and toggles between them in order to determine, visually, how well brain structures coincide in the different image sets.

The second evaluation method used anatomical landmarks to (1) evaluate quantitatively the registration quality, and (2) evaluate global distortions by analyzing the transformation matrix values. Seven anatomical landmarks were selected by an experienced neurosurgeon on each 1.5 T and 7 T MRI scans prior to image co-registration. Each landmark was characterized by a clearly defined and unique anatomical location in 3D space. The landmarks chosen for the T_1_W images were: 1) the ventricular surface of the anterior commissure at its rostro-caudal midpoint (AC), 2) the ventricular surface of the posterior commissure at its rostro-caudal midpoint (PC), 3) the rostro-caudal midpoint of the cerebral aqueduct, 4) the midpoint of the optic chiasm, and 5) the midpoint of the pituitary infundibulum. Additional landmarks were added on the cortical surface and sulci where a clear 3D location could be defined. In some cases, landmark #5 could not be identified due to lose of signal at the inferior frontal areas, near air cavities, where high susceptibility is present (see Discussion).

Landmark selection and identification on the T_2_W images was more challenging since it employed an axial non-isotropic acquisition (0.4×0.4×2 mm^3^) from the top of thalamus down through the upper pons slab (18–22 slices). The non-isotropic nature of this slab made it difficult to determine precise anatomical locations in 3D. Amira 5.3.3 software (Visage Imaging, Richmond, Australia) was used to interpolate and re-slice the T_2_W images in order to increase the accuracy while determining the 3D landmark location. The landmarks were chosen based on corresponding anatomical structures such as AC, PC, and clearly defined structures that were contained in one slice only.

Following landmark positioning, the global transformations that resulted from the above-mentioned registration (7 T to 1.5 T) were applied to the landmark coordinates of the 7 T images, transforming them into 1.5 T image space coordinates. Note that we used a landmark-free registration, involving validation based on anatomical landmarks. The transformed 7 T landmarks were visually evaluated for consistency with their corresponding 1.5 T anatomical landmarks. In addition, the 3D Euclidean distances between the landmarks of the 1.5 T images and the transformed landmarks of the 7 T images were calculated for each landmark. This allowed for a quantitative estimation of the *global* registration of the 7 T images. Figure 1 illustrates schematically the workflow of this process of evaluating the global registration quality using the landmarks method. The values for scaling, skews and determinant of the transformation matrices were evaluated in order to determine the amount of non-rigid transformation that was required for the registration.

**Figure 3:**
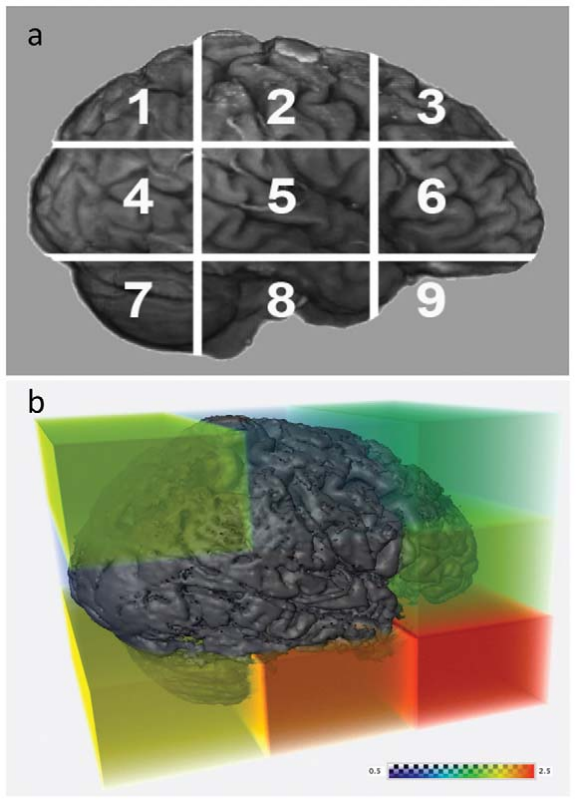
Regional contribution to global registration. **a**) Nine sub-volumes to which the T_1_W brain images were parceled for the local registration method. The regions are: 1. Posterior-superior, 2. Mid-superior, 3. Antero-superior, 4. Posterior-middle, 5. Middle (includes the midbrain and portions of the temporal lobes), 6. Antero-middle, 7. Posterior-inferior (corresponds mostly to the posterior fossa contents i.e., cerebellum), 8. Mid-inferior (corresponds primarily to the inferior portion and floor of the middle fossa) and 9. Antero-inferior (corresponds primarily to the anterior skull base and temporal poles). T_2_W images were parceled into three sub-volumes, which approximately correspond to the three middle regions of the T_1_W images (regions 4, 5 and 6). **b**) Average local skews of the parceled regions, superimposed on a surface rendering of a representative brain. The colors (cold-to-hot) reflect the amount of average regional skew required for a registration at that region (measured as the tangent of the skew angle). Note that distortions at the middle region, which include the midbrain and portions of the temporal lobes, are minimal and indicate the clinical applicability of 7 T imaging of these sub-volumes for DBS procedures for example. The registrations of the inferior regions (regions 7, 8 and 9) were typically unsuccessful due to loss of signal in these areas in the 7 T images, which did not allow meaningful estimation of distortion when using this method.

A third evaluation method was used in order to estimate the *regional* (per-region) contribution to the global degree of correspondence between the two sets of registered images. Following the global registration, T_1_W images of the brains were parceled into nine corresponding sub-regions as shown in Figure 3a. T2-weighted images were parceled into three sub-regions, which approximately corresponded to the three middle sub-regions of the T_1_W images (regions 4,5 and 6). Each 7 T sub-volume was then co-registered separately and independently, within its corresponding 1.5 T sub-volume, and the registration results were visually evaluated, where the success criteria for the registration was improving on the result of the global registration for that area [26]. The total regional transformation matrix was calculated by combining the resulting local transformation matrix with the global transformation matrix. The values of total scaling, skews, and transformation determinants for registration of each sub-volume were derived from the resulting transformation matrices and were recorded for each sub-volume. The scaling, skew, and transformation determinant required for the piecewise registration indicate, as a first order approximation, the amount of geometrical distortion of each region. The three most inferior regions containing the cerebellum, brainstem, and the orbital regions, were excluded from the analysis due to expected significant signal loss and susceptibility artifacts, resulting from proximity to air cavities and regions with large inhomogeneity.

A comparison was performed between the results of the distortion analysis of the two 7 T magnets (7T/PS and 7T/AS), as the latter is expected to present more challenges in terms of distortion correction, due to the employment of higher performing gradients with probably less overall uniformity.

## Results

A visual example of the registration results is shown in Figure 2, in which edges (red lines) obtained from the registered 7T/PS image are superimposed on the 1.5 T image. Two examples are shown: the top row depicts T_1_W coronal images acquired at (a) 1.5 T and (b) 7T/PS; the lower row depicts T_2_W axial images acquired at (c) 1.5 T and (d) 7T/PS, respectively. Similar image orientations as shown are used for planning the trajectory of the DBS electrode into the subthalamic nucleus (STN) for the surgical treatment of Parkinson's disease. The geometrical accuracy of these images is crucial to the success of any imaging-based stereotactic procedure. As suggested by this figure, there was a high degree of correspondence between the same anatomical features in the two image datasets, providing qualitative support for an adequate registration between the images.

**Figure 4:**
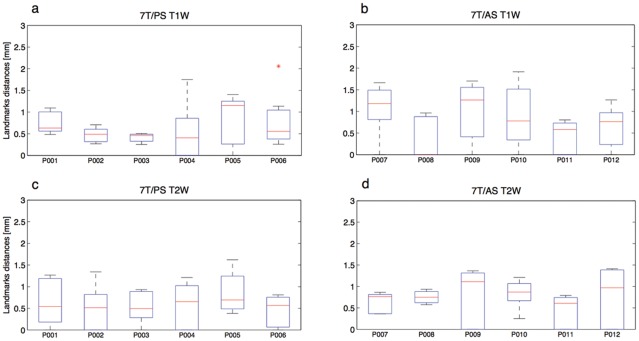
Quantitative and statistical summary of anatomical based registration. Box plots of the statistics of the co-registered landmarks' distances, obtained from individual patients, for T_1_W (top row – a and b) and T_2_W (low row – c and d) images. Further comparison is done between images acquired by 7T/PS (left column – a and c) and 7T/AS (right column – b and d). The red lines represent the average, top and bottom of the box represent the first and third quintile, respectively, the whiskers show the maximum and minimum values, and the asterix represent the outliers. The statistics is of distances between the anatomical landmarks assigned on 1.5 T images and the corresponding anatomical landmarks assigned on the 7 T images, after a global landmarks-free coordinates transformation to the 1.5 T image space. As can be seen, the distances are on the order of one voxel, indicating a high degree of correspondence between the two co-registered images.

The next level of analysis quantifies and characterizes the distortions and the degree of correspondence between the two sets of images using the anatomical landmarks. Figure 4 provides a quantitative summary of these findings, with boxplots showing the distribution of the distances between the selected anatomical landmarks, assigned on 1.5 T images, and their corresponding anatomical landmarks on the 7 T images. Each box represents the statistical distribution of the 3D Euclidean distances, obtained in the individual patients, for T_1_W (top row – a and b) and T_2_W (bottom row – c and d) images, respectively. We further compared the results of the 7T/PS MRI (a and c) and the 7T/AS MRI (b and d). Most distances were less than one voxel, indicating quantitatively high degrees of correspondence and a successful co-registration of the two image datasets. The results of the 7T/AS T_1_W images (4b) show greater distances between the corresponding registered landmarks (1±0.5 mm), likely resulting from differences in gradient linearities.

Outlier landmarks were observed mainly in inferior frontal areas (e.g., optic chiasm and the midpoint of the pituitary infundibulum), which, due to proximity to air-filled cavities, have higher susceptibility issues, resulting in local distortion and loss of signal. Images acquired from subjects P008 and P010 were notable for an excessive amount of motion during the acquisition, as a consequence of the patients' resting tremor, resulting in degraded image quality and more challenging registration and landmark identification. Nevertheless, the quality of the registration for datasets acquired from these two subjects was well within the range of error of one voxel for the landmarks.

**Figure 5:**
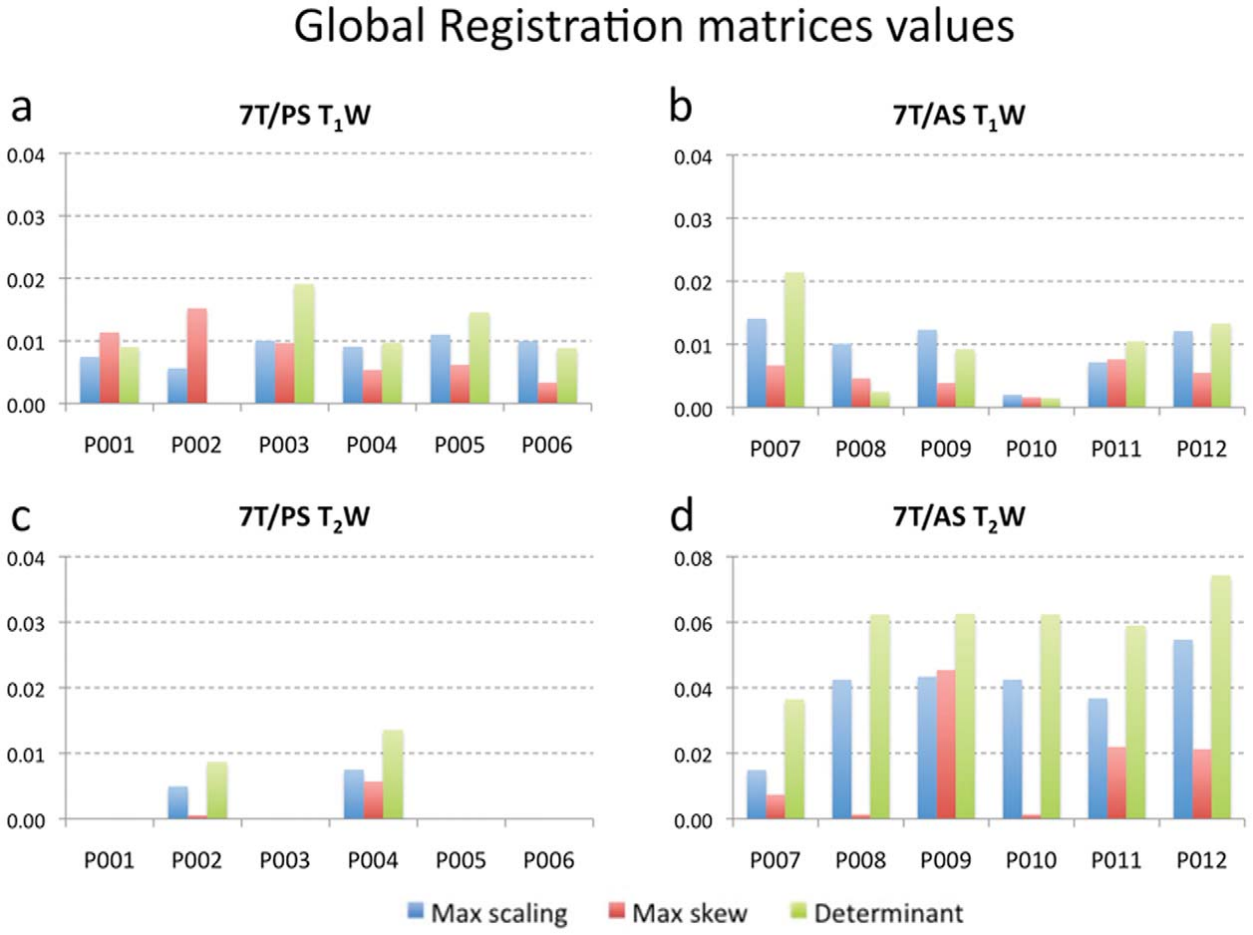
Global registration matrices values. Transformations values for the registration used for the T_1_W (top row – a and b) and T_2_W (low row – c and d) and for 7T/PS (left column – a and c) and 7T/AS (right column – b and d) images, as derived from the corresponding transformation matrices. The transformation values are measured as maximum scaling change (deviation of the scaling form 1), maximum skew (measured as the tangent of the skew angle), and volume change (deviation of the transformation determinant form 1) that were needed for global registration between 7 T and 1.5 T images. The values indicate that the registration operators are essentially rigid body transformations with negligible linear corrections in scaling, skew, and the transformation determinant. Given the high degree of correspondence between the registered images, these values indicate minimal amount of geometric distortion between the 1.5 T and 7 T images. Greater amount of scaling and skew was recorded for the 7T/AS T2-weighted images (5d). This is due to a stronger gradient employed in the 7T/AS MR system.

Figure 5 summarizes the transformation values for the co-registrations of T_1_W (top row – a and b) and T_2_W (lower row – c and d) images, as derived from the transformation matrices (see Methods). A comparison is shown between the results of 7T/PS (a and c) and 7T/AS (b and d). As the values indicate, the registration operators are essentially rigid body transformations with extremely small corrections in scaling and skew. The maximum scaling factors—i.e., those most divergent from 1—are on the order of a 1% change, and the maximum skews (maximum absolute skew value) are less than 0.01 (measured as the tangent of the angle). The transformation determinants, which relates the volume change of a region in the original image to the volume of the corresponding region after being transformed, are close to 1, meaning that essentially no volume change was required for registration. Transformation values for the T_2_W images suggest even lower distortions. In several datasets acquired on the 7T/PS, (i.e., P001, P003, P005 and P006), 6 DOFs were sufficient to get an accurate co-registration. In summary, the co-registration of both T_1_W and T_2_W images between 7T/PS and 1.5 T datasets required basically rigid body transformation.

A larger amount of scaling, skew and volume change was required for the 7T/AS T_2_W image registration (Figure 5d). This was not unexpected, as discussed below.

**Figure 6:**
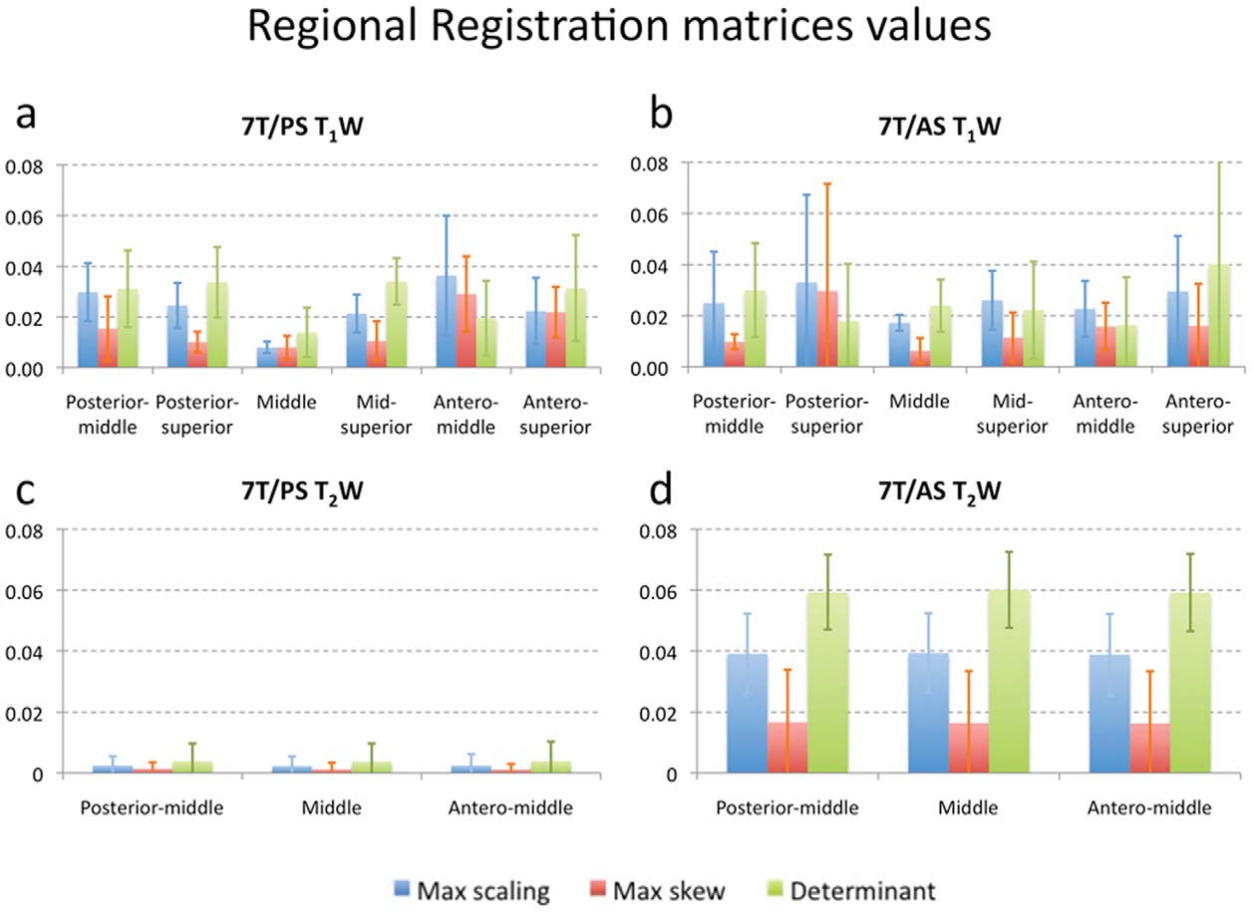
Regional registration matrices values. Average values (per brain region) of maximum scaling change (measured as the scaling deviation form 1), maximum skew (measured as the tangent of the skew angle) and volume change (measured as the deviation of the transformation determinant form 1), that were needed for regional registration between 7 T and 1.5 T images. Presented are the values of T_1_W (top row – a and b), T_2_W (low row – c and d), 7T/PS (left column – a and c) and 7T/AS (right column – b and d). These values represent first order approximations of the amount of distortion associated within each part of the imaging volume. As expected, the Posterior-superior, Antero-superior and Antero-middle, exhibit greater distortion, while central and mid-back regions presented minimal levels of distortion.

The third level of analysis involved an evaluation of the degree of correspondence, on a *regional* basis, between the 7 T and 1.5 T images. These results are summarized in Figure 6, showing a comparison between T_1_W (a and b) and T_2_W (c and d), and between 7T/PS (a and c) versus 7T/AS (b and d). The values in Figure 6 depict a first-order approximation of the amount of distortion associated with each part of the volume data. As expected, the posterior-superior, antero-superior and antero-middle sub-regions (regions corresponding to areas 7, 8, & 9 in Fig. 3a), were associated with the largest deformations, while the middle sub-region, which included the midbrain and portions of the temporal lobes, required minimal corrections. A minimal amount of correction was observed in the 7T/PS T_2_W images (Fig. 6c) compared with the amount needed for the 7T/AS T_2_W images (Fig. 6d). This finding is likely a consequence of the difference in gradient design between the 7T/AS and 7T/PS systems, as further discussed below. Figure 3b illustrates the nine sub-regions into which the brain was divided, superimposed on a surface rendering of a representative brain. The colors are a heat map reflecting the average amount of regional skew that was required for each region during the registration of the T_1_W 7T/PS datasets (measured as the tangent of the skew angle). The registration of the most inferior regions (Posterior-inferior, Mid-inferior and Antero-inferior) was typically unsuccessful due to signal loss in these areas in the 7 T images, which did not allow for a meaningful estimation of distortion when using this (regional registration) method. Therefore, only data acquired for the six upper regions are presented.

## Discussion

In this work, we qualitatively and quantitatively estimated the degree of correspondence between ultra high-field (7 T) MR imaging and clinical 1.5 T MR imaging within a realistic clinical setting. The results of this investigation demonstrate that direct clinical applications of 7 T MRI are feasible.

Geometric distortions in MR images can originate from (a) MRI system imperfections, and from (b) subject-dependent sources. The former include gradient nonlinearities, inhomogeneity in constant magnetic fields (B0), and imperfect shimming. The primary subject-dependent sources include chemical shift artifacts and susceptibility effects which are enhanced at higher magnetic field strength [27].

In this work, an affine registration was used as a tool to estimate and characterize possible geometrical distortion. The first stage of the analysis involved achieving an acceptable level of co-registration and then verifying the adequacy of the co-registration both qualitatively (iso-contours/edges method) and quantitatively (anatomical landmarks method). Following this co-registration, the analysis of the transformation matrices enabled detection of the presence, and estimation of the amount, of geometric distortion between the co-registered image datasets.

As shown in the Results section, an affine transformation already allowed for high quality registrations, both qualitatively and quantitatively. Quantitative evaluation of the registration showed that the error associated with the co-registration of 7 T MR images to 1.5 T MR images is on the order of 1 mm, which is comparable to the intrinsic error of approximately one voxel, expected from the technique used for estimating the registration quality (see Error Analysis section). The error in the registration of the 7T/AS T_1_W images (Figure 4b) appears to be slightly greater. This result is expected as the 7T/AS employs a different gradient design intended to allow higher performance, which may suffer from increased nonlinearities, for which compensation is more difficult. Nevertheless, the registration error is still well within the sub-millimeter range.

The overall T_2_W image registration error was significantly less than 1 mm. This might be in part due to imaging only the mid-part of the brain, thereby avoiding regions of higher susceptibility artifact and gradient non-linearity.

Few outliers were observed for which the distances between corresponding anatomical landmarks varied from 2 mm to 4 mm (see Figure 4). As expected, these landmarks were situated in inferior frontal areas, adjacent to air-filled sinuses (e.g., optic chiasm and pituitary body), where higher susceptibility causes greater local distortion as well as signal loss. Clinical applications aimed at targets in the inferior frontal region will need to take into account this propensity for greater distortion, and methods for mitigating the distortion will need to be developed, e.g., by considering non-linear registration techniques or simply limiting the use of 7 T in those particular sub-regions. However, these local nonlinear distortions did not affect DBS targets in the region of the basal ganglia or thalamus.

The next stage involved analyzing the transformation matrices in order to estimate and characterize the distortions. An affine registration process as the one studied here can be divided into three types of transformations: (1) rigid body, (2) scaling, and (3) shearing. A rigid body transformation is merely a change in the location and position of the object being imaged. Performance of this type of transformation does not indicate the presence of any distortion. Scaling indicates only a change in the focal point. This, we suggest, can be regarded as a weak form of distortion, as it does not change the geometrical relationship between different points (regions) in the image and does not change the overall planning of clinical applications once accounted for (as long as the image scale is known or can be estimated). In contrast, shearing *must* be regarded as a real geometrical distortion, and it likely originates from the nonlinearities of the gradients. Nevertheless, as long as image co-registration through the use of an affine transformation (for example) is possible, other imaging modalities which are less susceptible to geometric distortions, such as CT [28], [29], can be used to transform the high-field image into the true patient coordinates, without losing any information or the integrity of the data.

As summarized in Figure 5, analysis of the transformation matrices demonstrates minimal amounts of scaling, skew, and volume change. This finding, together with the observed, quantitatively measured, adequate registration, is strong evidence that there are minimal geometric distortions in the 7 T images compared to the clinical 1.5 T images in regions relevant for surgical planning of DBS.

**Figure 7:**
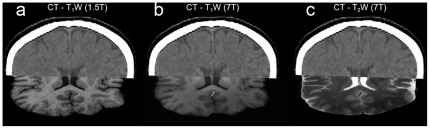
Registration of 7T MRI to CT. An example of co-registration between CT and a) 1.5 T T_1_W, b) 7 T T_1_W, c) 7 T T_2_W. Although CT images are far less informative than MRI, it can be seen that the ventricles are perfectly aligned, indicating an adequate quality of registration. Registering 1.5 T MR images to a CT is a common practice for planning of neurosurgical procedures including DBS surgery and tumor resections. This practice allows for using the superior contrast of MRI while capitalizing on the geometric integrity of the CT image. Here we suggest that the same practice may be used for correcting for whatever geometrical distortions that may be present in 7 T MRI.

A larger amount of scaling, skew, and volume change was observed in the 7T/AS T_2_W transformation matrices. A detailed analysis of the origin of these changes reveals a significantly larger scaling in the cross-plane (gradient Z) direction (inferior-superior human axis) and significantly more skew on the YZ plane (sagittal plane) compared with the 7T/PS MRI system. Given the fact that the gradients used in the sequence and the sequence parameters (bandwidth, etc), are identical for both 7 T scanners, this difference in distortion is most likely due to the larger known gradient nonlinearities in the 7T/AS MRI systems, which are harder to be compensated for by the distortion correction algorithm that is employed by the MRI manufacturer. These non-linearities depend only on the physical characteristics of the gradient hardware. Nevertheless, as can be deduced from the registration results, these distortions can be effectively compensated for by a simple affine registration to a modality less susceptible to distortion, such as CT. Note that registering clinical 1.5 T MR images to CT images in order to compensate for distortions in the 1.5 T MR images is also common practice for neurosurgical planning. To further emphasize the ability of using CT to correct for the geometrical distortions of 7 T MR images, we followed the clinical procedure and co-registered 1.5 T and 7 T MR images to pre-operation baseline CT images in five representative cases. Figure 7 depicts one such example and illustrates the quality of co-registration between pre-operation CT and a) 1.5 T T_1_W, b) 7 T T_1_W and c) 7 T T_2_W. Although CT images are far less informative than MRI, it can be seen that the ventricles are aligned, indicating an adequate quality of registration. These co-registrations were confirmed by an experienced neurosurgeon (AA) to be sufficient for use in the operation room for navigational planning.

As the results suggest, the distortion in these regions, with the imaging protocols used, are not dominated by high-field effects, but more likely by gradient non-linearity effects. This can be deduced by comparing the results of the 7T/PS to the results of the 7T/AS. Using more conventional gradients, or improving the distortion corrections of the gradients, will result in smaller geometrical displacements or distortions as depicted by the 7T/PS results (Figure 5c).

Finally, it should be noted that we do not claim that the registration algorithm used here is optimal. It is possible that an optimized (non-linear) registration algorithm would lead to even better results. We used only a conventional registration algorithm that is widely used in clinical settings as a tool (1) to obtain a quantitative estimate of geometrical distortions, and (2) to demonstrate that compensation for this distortion is achievable, even with such simple, but clinically plausible techniques.

Further analysis was performed by independently registering selected sub-regions of the images, and estimating the spatial distributions of the local distortions. As the results summarized in Figure 6 show, the distortion of the 7 T MR image was distributed non-uniformly. Generally, the images tend to be more distorted at parts located further away from the gradient center, where correcting for the non-linearity of the gradient field is more challenging (and might call for non-linear methods). This is apparent in the local registration of the T_1_W images, where peripheral regions of the brain present more scaling, skew, and volume changes than the central regions. Analysis of co-registration of the middle part of the brain demonstrated minimal amounts of distortion, which is an encouraging result for clinical applications that involve imaging this region, such as targeting STN, VIM, or the internal segment of the globus pallidus (GPi) during DBS surgery.

Analyzing the results of the regional registration of the T_2_W 7 T images (figures 6c and 6d), further supports the claim that minimal distortion occurs in the middle slab of the image, close to the center of the gradient. Comparing the results from the global registration to the local registration shows that almost no correction was required for the global registration, resulting in similar transformation matrices values.

Local registration of each sub-region within the brain requires some fine-tuning of the registration process. Registration of each sub-region within the brain may result in better local registrations as a consequence of relaxing the constraint that requires the whole brain to be properly registered. However, relaxing these constraints might also result in reducing the robustness of the registration process and potentially increasing the overall registration error (e.g., clear corresponding areas outside of a sub-region might help in the registration of the sub-region itself). The large error bars in the values of the local transformation matrices (Figure 6) were a result of such a loss of robustness. Consequently, in cases where the local sub-region registration failed, additional strategies were employed to improve the results (e.g., limiting the searching angle, changing the cost function to correlation ratio, and trying different initial conditions). It is again worth noting that optimizing the registration method was not the aim of this work, and results obtained here would likely improve using more advanced techniques.

### Error analysis

The error of the landmark-based method for evaluating the registration quality and estimating the geometric distortion originates from three main sources: First, landmark placement is subject to the accuracy of the human eye, experienced as it may be (in our case, they were placed by a neurosurgeon). To minimize this source of error, only clear and relatively discrete locations in 3D space were selected for landmark placement. Since the size of the anatomical structures used as landmarks were approximately 1 mm, and the landmark marker was 0.5 mm in diameter, we estimate the resulting marking error to be approximately 0.5 mm in size. We have validated this estimation by reanalyzing the entire data from two patients, in which we have repeated the landmarks selection and computed the overlap between the earlier and current landmark positions. On average, the two sets of landmarks diverged by 0.61 mm, confirming our estimation. Second, the image resolution itself poses an intrinsic limit to the accuracy of the landmark placement. Sub-voxel structures are spatially averaged (partial-volume effect), and therefore landmarks often cannot be placed with accuracy of less than one voxel in size. Selecting structures with high contrast-to-noise ratio (CNR) allows the use of interpolation in order to enhance the accuracy of the landmark placement. Both sources of landmark placement error are highly correlated and therefore the total expected error is estimated to be one voxel. Note that in the case of anisotropic voxels, the error size is anisotropic as well. For example, while the in-plane error of the T_2_W images is 0.4 mm, the cross-plane error is approximately 2 mm, which is same as the slice thickness. In order to mitigate the cross-plane error, it is possible to acquire slices in the coronal orientation of the region-of-interest (ROI) and fuse these with the axial view in order to get a higher resolution image of the ROI [30]. Note that such errors in landmark location are also part of the current standard clinical practice, where manually selected landmarks are used to register the patient images to consensus coordinates.

Finally, the registration algorithm does not guarantee that the resulting transformation is the best possible. Different choices of degrees-of-freedom, cost function, interpolation method, search angles, and optimization method, may result in different (and possibly improved) outcomes. Nevertheless, finding an affine transformation that maps one set of landmarks (7 T) to the other (1.5 T), sets an upper limit on the amount of expected distortion, at least for the regions covered by these landmarks. As demonstrated here, the upper limit obtained with these simple techniques is already extremely low.

### Conclusion

The transformations that were used here for co-registration of 7 T to 1.5 T MR image datasets were essentially rigid body rotations and translations provided that all non-brain tissues (e.g., skull, neck, nose) were removed. Our ability to register images from a 7 T magnet to images from a 1.5 T magnet, using rigid body transformations, suggests that geometrical image characteristics of ultra-high field MRI are comparable to those of images acquired on a clinical 1.5 T MR system, at least with the imaging protocols used and for the regions investigated here - region housing current FDA-approved targets for the treatment of movement disorders and are relevant for surgical planning in DBS.

It is not our intention to state that there was no geometrical distortion present. We simply suggest that the distortions currently seen on 7 T MRI are comparable with distortions observed on 1.5 T MRI in a clinical setting. Nevertheless, 1.5 T MR images are routinely used for planning of neurosurgical procedures including DBS surgery and tumor resections, despite the fact that 7 T can provide significantly improved visualization [8], [9]. This research suggests that the development of ultra-high field instrumentation has progressed to a point where the benefits of higher resolution and better contrast-to-noise (CNR) afforded by high-field MRI are clinically feasible. Our finding that essentially only affine transformations are mandatory for sufficient co-registration between 1.5 T and 7 T images imply that minor changes in conventional platforms' (e.g., Stealth) algorithms and/or protocols may be sufficient to allow 7 T data to be utilized in the clinic. Furthermore, preliminary and ongoing research based on the 7 T to 1.5 T registration reported here, suggests that 7 T images can improve targeting objectives in the clinical setup during DBS surgery (manuscript in preparation). In addition, future research could capitalize on the enhanced resolution of high-field imaging and explore the topic of distortions associated with intracranial processes, resulting in brain shifts, and their impact on direct targeting.
